# Autologous erythrocytes delivery of berberine hydrochloride with long-acting effect for hypolipidemia treatment

**DOI:** 10.1080/10717544.2020.1716880

**Published:** 2020-02-03

**Authors:** Zhongyao Cheng, Siyu Liu, Xinyi Wu, Faisal Raza, Yichen Li, Weien Yuan, Mingfeng Qiu, Jing Su

**Affiliations:** School of Pharmacy, Shanghai Jiao Tong University, Shanghai, China

**Keywords:** Red blood cells, berberine hydrochloride, biocompatible, sustained, hypolipidemic

## Abstract

Discovery of novel pharmacological effects of berberine hydrochloride (BH) has made its clinical application valuable. However, further development and applications of BH are hampered by its short half-life and the side effects associated with its intravenous (iv) injection. To improve the hypolipidemia efficacy and reduce side effects, we encapsulated BH into biocompatible red blood cells (RBCs) to explore its sustained-release effect by hypotonic pre-swelling method. From *in vitro* evaluation, BH loaded RBCs (BH-RBCs) presented similar morphology and osmotic fragility to native RBCs (NRBCs). After the loading process, the BH-RBCs maintained around 69% of Na^+^/K^+^-ATPase activity of NRBCs and phosphatidylserine externalization value of BH-RBCs was about 26.1 ± 2.9%. The survival test showed that the loaded cells could circulate in plasma for over 9 d. For *in vivo* evaluation, a series of tests including pharmacokinetics study and hypolipidemic effect were carried out to examine the long-acting effect of BH-RBCs. The results showed that the release of BH in the loaded cells could last for about 5 d and the hypolipidemic effect can still be observed on 5 d after injection. BH-loaded autologous erythrocytes seem to be a promising sustained releasing delivery system with long hypolipidemic effect.

## 1. Introduction

Berberine hydrochloride (BH) is a well-known bioactive alkaloid widely used for diarrhea commercially. It has few oral adverse effects and is the first-choice treatment for bacillary dysentery (Liu et al., 2016). Recent research has demonstrated that BH also possesses other therapeutic use such as anti-malaria, anti-hypertension, anti-hyperlipidemia, anti-arrhythmia, anti-tumor, hypolipidemic and neuroprotective effects (Tan et al., 2011; Chang et al., 2015; Zhou et al., 2016; Zou et al., 2017; Poudel et al., 2019). Despite its therapeutic effects, the clinical use of BH has been limited due to its low bioavailability and short half-life. Besides, it can be quickly distributed into organs and tissues after intramuscular and iv administration, which cause various side effects such as vasodilatation, hypotension, cardiac inhibition, and so on (Yu et al., 2017). Since its oral dosage forms and iv injection preparations have limited its therapeutic applications, there is an intense need to develop new delivery systems to overcome the above-mentioned problems.

To prolong the release and increase the bioavailability of BH, the administration of drug encapsulated in biodegradable polymeric nanoparticles has been investigated. For example, solid lipid nanoparticles (Wang et al., 2017), gold nanoparticles (Yu et al., 2016), and so on. Despite these, the reported nanodosage forms of BH still have low oral bioavailability and many side effects that need to be overcome (Su et al., 2019). Furthermore, considering that BH is currently being developed for the treatments of cardiovascular disease and cancer, which require long-term administration of drugs. It is important to note that frequent administration often leads to poor patient compliance. At the same time, the rapid clearance from circulation by the mononuclear phagocyte system was also reported (Luk et al., 2013; Shi et al., 2014; Luk & Zhang, 2015). Thus, a more biocompatible vector for BH loading is strongly needed.

RBCs are the most abundant type of blood cells which function as natural carriers for oxygen and have a life span of 100–120 d (life-time of mouse RBCs is about one-third of human’s counterpart) (Muzykantov, 2010). Compared with other carriers, RBCs serve as an ideal candidate for drug delivery with long circulation time, excellent biocompatibility and low immunogenicity (Lopez et al., 2017; Sun et al., 2017). The crucial self-markers covered on the surface allow RBCs to circulate *in vivo* for a long period without being cleared by macrophages (Oldenborg et al. 2000; Hamidi et al., 2007). As reported by Rossi et al. (2001), dexamethasone 21-phosphate, a therapeutic agent for chronic obstructive pulmonary disease (COPD) was loaded inside RBCs. The concentration of dexamethasone in blood after single administration of drug-loaded erythrocytes to patients with COPD was maintained for ≤7 d. Multiple strategies have been developed to load RBCs with cargoes, including electric pulses (Zhou et al., 2010) and hypotonic dialysis (Sun et al., 2016).

**Figure 1.**
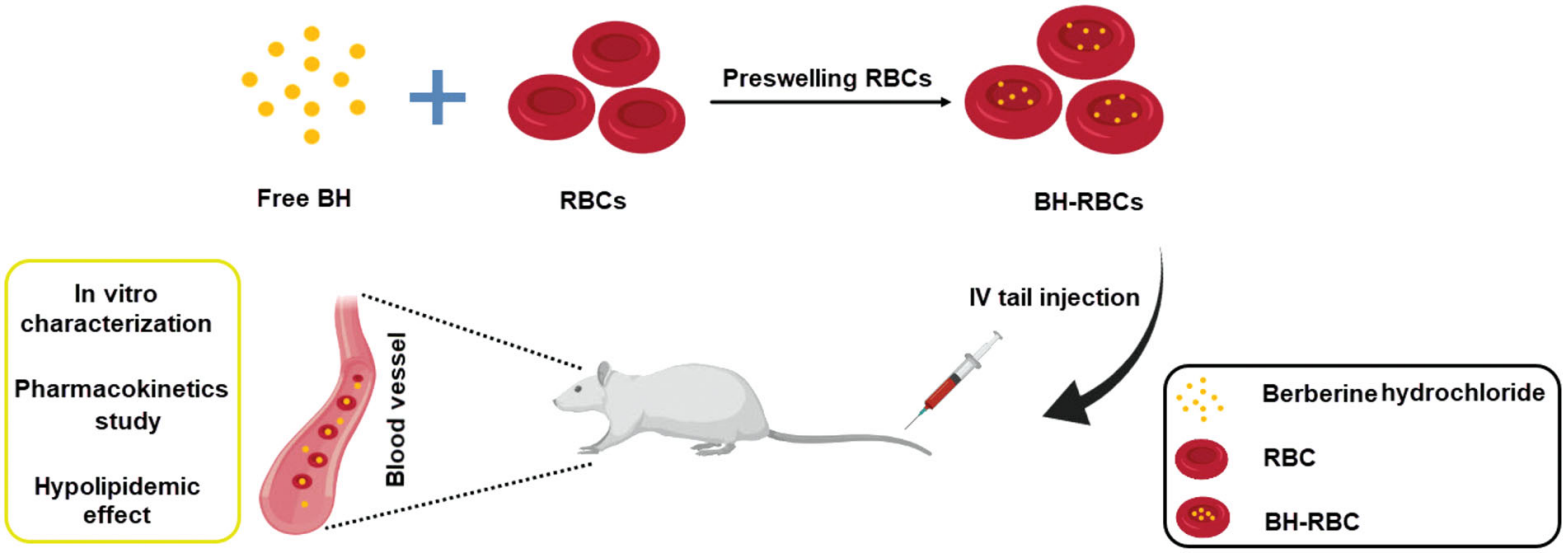
Schematic illustration of the preparation and characterization of BH-RBCs.

In this study, BH was loaded inside RBCs using hypotonic preswelling method (Figure 1). To examine the long-acting effects of loaded cells, survival of the BH-RBCs in circulation, drug release in plasma and anti-hypolipidemic effect after injection were investigated. Altogether, the developed BH-RBCs preparation showed sustained releasing property of BH. From our results, the BH-RBCs are promising delivery system that can achieve long circulation and sustained-release of BH.

## 2. Materials and methods

### 2.1. Materials

Berberine hydrochloride (HPLC ≥ 99%) and tetrahydropalmatine were purchased from Meilun Biologics (Dalian, China). Sodium bicarbonate was obtained from Macklin (Shanghai, China). Glutaraldehyde solution (50 wt% in H_2_O) and glucose were purchased from Shanghai Titan Technology Co., Ltd. (Shanghai, China). Methanol at HPLC grade was purchased from Merck & Co., Inc. (Kenilworth, NJ). PBS (phosphate-buffered saline, 500 mL) and FITC (fluorescein isothiocyanate, 10 mg) were purchased from Tianjin Biolite Biotech Co, Ltd (Tianjin, China). All other chemicals were of analytical grade. NHC-LC-Biotin reagent was purchased from APExBIO Company (Houston, TX). FITC streptavidin was obtained from Nanjing Xinfan Biological Technology Co., Ltd. (Jiangsu, China) BCA Protein Quantification Kit was purchased from BOSTER Biological Technology, Wuhan, China. Na^+^/K^+^-ATPase Kit, Annexin V-FITC kit were both purchased from Nanjing Jiancheng Bioengineering Institute, Jiangsu, China.

### 2.2. Animals

SD (Sprague–Dawley) rats (weighing about 200 g) and C57BL/6 mice (8-week-old males weighing about 20 g) purchased from Beijing Charles River Co., Ltd (Beijing, China) were used in the project. All procedures performed in studies involving animals were in accordance with the Guidelines for Care and Use of Laboratory Animals of Shanghai Jiao Tong University and approved by the Animal Ethics Committee (Number: A2019033).

### 2.3. Preparation of BH-RBCs

The whole blood was collected from rats by orbital plexus vein and transferred into a tube with heparin sodium for anticoagulation. Then centrifuged at 4 °C, 600×*g* for 3 min to remove the plasma and leukocytes in the supernatant, and washed three times with 5% isotonic glucose solution at 4 °C. A pre-swelling method was used for loading BH into RBCs (Harisa et al., 2014). 100 μL washed packed RBCs were added to 1 mL hypotonic drug solution (5.2 mg/mL NaHCO_3_, 2.5 mg/mL BH after heating), incubated at room temperature for 20 min. Gentle mixing was done every 4 min. Then 0.2 mL hypertonic solution (60 mg/mL NaHCO_3_) was added to the system, mixed gently and held for 30 min to reseal erythrocytes. The sample was centrifuged and supernatant was discarded. Then washed with 5% isotonic glucose solution for 3 times to remove the free drug and obtain drug-loaded RBCs.

### 2.4. Drug loading contents

The BH content in BH-RBCs was determined by HPLC. 1 mL pure water was added to the BH-RBCs preparation, standing for 5 min to lyse the drug-loaded RBCs completely, and then centrifuged to remove the supernatant. Before determining, 10 times methanol was added into supernatant and vortex for 1 min to precipitate the hemoglobin, then the supernatant 2 after centrifuging at 3000 *g* for 10 min was filtered and 20 μL filtrate was injected into HPLC system automatically.

Separations were performed on the Agilent SB-C18 column (4.6 mm × 250 mm, 5 μm), and detection was monitored by an ultraviolet detector at 345 nm for BH. The mobile phase for BH was acetonitrile with 0.05 mol/L KH_2_PO_4_ solution containing 0.4% (w/v) sodium dodecyl sulfate (adjusting the pH value to 4 by adding phosphoric acid) (50:50, v/v). A flow rate of 1.0 mL/min was used:

Drug   loading   amount  (mg/mL cells) = Entrapped   amount   of   drug/0.1 mL   cells.

### 2.5. Scanning electron microscopy (SEM)

Two types of RBCs for native RBCs (NRBCs) and BH-RBCs were added to an EP tube containing 1 mL 2.5% glutaraldehyde to fixing for 30 min, then centrifuged to remove the supernatant and incubating in an EP tube containing 1 mL post-fixation solution (0.4% potassium permanganate and 0.6% potassium dichromate), gently shaken and mixed for 5 min. The sample was then dehydrated at a concentration gradient of ethanol from 30% to 100%, with each concentration for 5 min. Finally, replaced by isoamyl acetate for 30 min (Fassett et al., 1983). The prepared samples were analyzed using a scanning electron microscope (SEM) after being coated with gold particles by a Sputter Coater in 18 mA for 1 min.

### 2.6. Osmotic fragility

The osmotic fragility test was performed to detect structural changes in RBC membranes subjected to osmotic stress. The fragile cells may be destroyed and eliminated quickly from circulation by macrophages (Hamidi et al., 2007). To evaluate the resistance of RBC against the hypotonic solutions media, 0.1 mL cells was added into 1 mL sodium chloride solutions with a concentration gradient (0.9, 0.8, 0.7, 0.6, 0.55, 0.5, 0.45, 0.4, 0.3, 0.2, 0.1, and 0.0% NaCl). After incubation for 30 min at 4 °C, the suspensions were collected by centrifugation at 600×*g* for 3 min. Then 100 μL the supernatant was added to a 96-well plate. Three parallel wells were set for each sample, and the absorbance at 540 nm of each sample well was measured by ultraviolet spectrophotometer. The hemolysis rate was calculated according to the following formula. A_540_ (308) means absorbance of sample in 0.9% NaCl solution equals to osmotic pressure of 308 mOsmL^−1^ and A_540_ (0) means absorbance of sample in 0% NaCl solution equals to osmotic pressure of 0 mOsmL^−1^. Draw an osmotic fragility curve:

$$\text{Hemolysis}\;\text{rate}\;\left(\%\right)=\frac{A_{540}-A_{540}(308)}{A_{540}\left(0\right)-A_{540}(308)}\times 100$$

### 2.7. Activity of Na^+^/K^+^-ATPase

To investigate the possible damages of loading procedure on RBC bioactivity (Jagadish et al., 2017), the activities of Na^+^/K^+^-ATPase of NRBCs and BH-RBCs were determined by Na^+^/K^+^-ATPase Kit. The RBC pellets were lysed in distilled water for 10 min. 20 μL RBC lysate was collected and diluted 50 times with distilled water until the lysate was colorless or slightly pink. Then the activity of Na^+^/K^+^-ATPase was measured according to the protocol of Na^+^/K^+^-ATPase Kit and protein concentration was determined using the BCA Protein Quantification Kit.

### 2.8. Phosphatidylserine (PS) exposure

Phosphatidylserine located on the inner layer of the cell membrane is the signal of apoptosis (Fadok et al., 1992). Therefore, the eversion of PS is often used as a signal to detect apoptosis. Cells with high PS values tend to be cleared more easily by the immune system (Staedtke et al., 2010). 10 μL RBCs were resuspended in 490 μL isotonic 1 × PBS firstly. Then 10 μL the diluted RBCs were resuspended in 100 μL 1 × binding buffer and incubated with 5 μL FITC-labeled annexin V for 10-15 min at room temperature in the dark. After incubation, 400 μL 1 × binding buffer was added to terminate the labeling reaction. The samples were analyzed by a flow cytometer (FACScan) and software (CELLQUEST, Becton Dickinson, Shanghai, China). PS reversion rate is the ratio of FITC labeled RBCs.

### 2.9. Survival in circulation

To study the *in vivo* circulation time of drug-loaded cells, BH-RBCs were labeled with biotin reagents and administered to the rats (Mock et al., 2011). The blood was taken at different time points and the survival rate of RBCs was studied by Flow Cytometry (Donnenberg et al., 2019). Briefly, the prepared BH-RBCs and NRBCs were mixed with biotin reagents (15 μg/mL) at ratio of 1 to 100 and incubated at room temperature for 30 min. The supernatant was removed by centrifugation and BH-RBCs were washed three times with isotonic 100 mM glycine PBS solution to terminate the biotin process. Finally, the biotin-RBCs were resuspended with autologous plasma and administered to rats by tail vein injection. 50 μL blood from rats was collected at different time points (5 min, 1 h, 3 h, 5 h, 8 h, 24 h, 3 d, 5 d, 7 d, and 9 d). After centrifugation and being washed with PBS, 10 μL packed erythrocytes were added to 0.5 mL PBS and 0.05 mL FITC-streptavidin solution. These were incubated for 30 min with shaking and protected from light. The supernatant was removed by centrifugation and washed twice with PBS. Finally, the RBCs were diluted 2000 times with PBS to measure the average fluorescence intensity of each point, and the average fluorescence intensity of 5 min was calculated as 100% survival rate. The survival rate of drug-loaded cells at each time point was calculated and the survival curve was plotted.

### 2.10. Pharmacokinetics (PK) study

The PK of BH in preparations was determined by UPLC-MS (Kim et al., 2019). 12 SD rats (about 200 g) were randomly divided into two groups. Rats were fasted 24 h before dosing and given free access to water. First, 1 mL the free BH solution and BH-RBCs (5 mg/kg) preparations were injected to rats respectively through the tail vein. Then 0.1 mL blood was collected by eye puncture method at different time points (5 min, 15 min, 30 min, 1 h, 2 h, 4 h, 6 h, 8 h, 12 h, 24 h, 2 d, 3 d, and 5 d). Samples were centrifuged at 13,200 rpm, 4 °C for 10 min. Then 30 μL plasma and 270 μL tetrahydropalmatine methanol standard solution (100 ng/mL) were mixed and vortexed for 10 min. Then samples were centrifuged again and the supernatant was analyzed by UPLC-MS. Standard PK parameters for BH were calculated by Pksolver version 2.0.

### 2.11. Safety test

The safety test of free BH solution and BH-RBCs was performed using C57BL/6 black mice (Anwar et al., 2018). 24 male mice were randomly divided into four groups. Mice were administered formulations after regular feeding for 3 d. 0.2 mL BH-RBCs (1 mg/mL) preparation and saline solution were administered to three groups by tail vein injection every 5 d for 3 consecutive doses (0 d, 5 d; 10 d). The mice in free BH group were injected with 0.2 mL BH solution (200 μg/mL) every day for 15 d. After 15 d, the mice were subjected to eyelid blood sampling to measure blood routine. The main organs of the mice (heart, liver, spleen, lung, and kidney) were separated for visually observed, weighed and tissue section analysis. After being washed with saline, they were fixed with 4% paraformaldehyde and embedded in paraffin blocks. The tissues were then sliced and H&E staining. Each tissue section was observed and image was captured.

### 2.12. *In vivo* hypolipidemic effect

The hypolipidemic effect of preparations was evaluated using C57BL/6 black mice hyperlipidemia model (Zhu et al., 2018; Raju et al., 2019). The male mice were randomly divided into four groups (*n* = 6), which were negative group, control group, BH-RBCs group and free BH group. The negative group was fed with normal diet and the other three groups were fed with high-fat diet for 2 months for modeling, then blood samples were taken from the orbital plexus vein to determine whether the blood lipid levels reached the hyperlipidemia model. After successful modeling, 0.2 mL saline solution (0.9%) was administered to negative and control group and 0.2 mL BH-RBCs (1 mg/mL) preparations were administered by tail vein injection every 5 d with 6 consecutive doses (0 d, 5 d; 10 d; 15 d; 20 d; 25 d). All mice in the free BH group were injected with 0.2 mL BH solution (200 μg/mL) every day for one month. The weight of the mice was measured before and after administration to determine the effect of various preparations preliminarily. After 30 d, serum was collected to determine the four levels of blood lipids (tiglyceride (TG), total cholesterol (TC), high-density lipoprotein cholesterol (HDL-C) and low-density lipoprotein cholesterol (LDL-C)). The difference of blood lipid levels was observed between the drug-administered group and the model group;

### 2.13. Statistical analyses

Data were presented as the mean ± SD from three independent experiments. The statistical significance of differences was analyzed by the Tukey Kramer multiple comparison tests, using GraphPad Prism Software, v.6.01 (GraphPad Software, Inc., La Jolla, CA). *p* < .05 was considered statistically significant.

## 3. Results and discussion

### 3.1. Drug loading contents

The loading contents of the prepared BH-RBCs was about 2.51 ± 0.26 mg/mL cells, which is a promising contents for the treatment of hypolipidemia (Higaki et al., 2005).

### 3.2. Scanning electron microscopy

**Figure 2.**
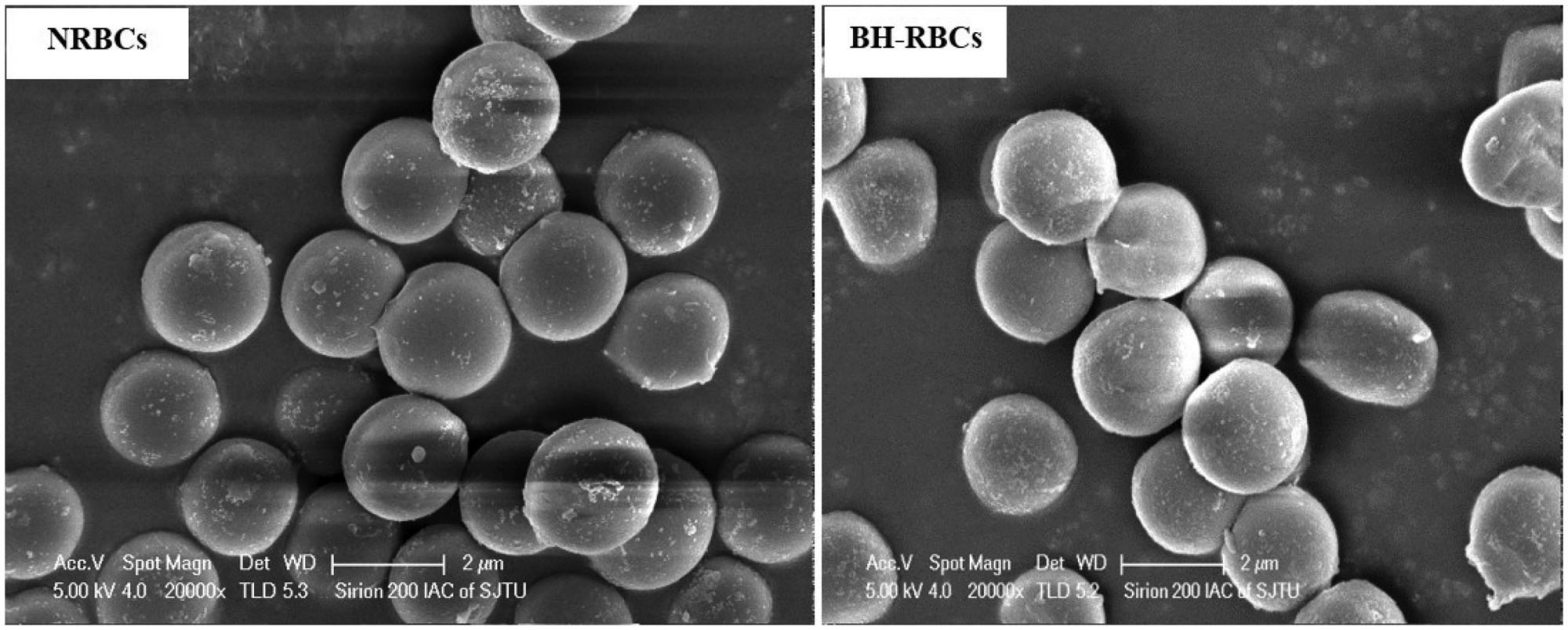
SEM of NRBCs and BH-RBCs. The magnification is 20,000.

The morphology of the erythrocytes reflects the damage degree during drug loading process We observed the possible morphological changes of erythrocytes upon loading process by using SEM. As shown in Figure 2, NRBCs have a normal biconcave shape and the morphology of BH-RBCs was similar to the NRBCs. The finding showed that the loading process might have little significant effect on erythrocyte’s shape.

### 3.3. Osmotic fragility

**Figure 3.**
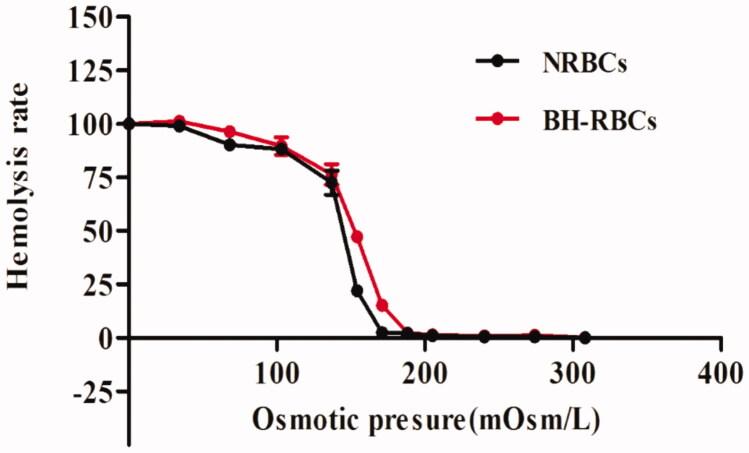
Osmotic fragility curves of NRBCs and BH-RBCs. Data are represented as mean ± SD (*n* = 3).

We monitored the osmotic fragility of the NRBCs and BH-RBCs. As shown in Figure 3, the initial hemolysis point and the complete hemolysis point of BH-RBCs and NRBCs is almost same. However, compared with NRBCs, the hemolysis rate of BH-RBCs is increased between 154 mOsm/L and 205 mOsm/L. BH-RBCs is more likely to be ruptured than NRBCs under the same osmotic pressure environment, indicating that a certain degree of damage to RBCs was caused during the drug-loading process *in vitro*, which is consistent with reports in related literature (Tajerzadeh & Hamidi, 2000). In all, the gap between them is acceptable.

### 3.4. Activity of Na^+^/K^+^-ATPase

**Figure 4.**
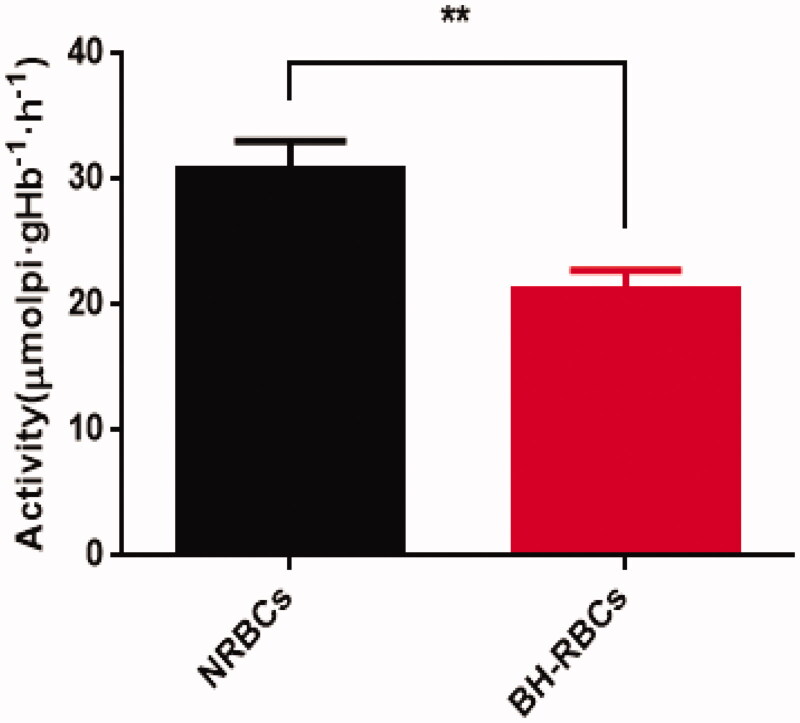
Na^+^/K^+^-ATPase activity on the membrane of NRBCs and BH-RBCs. Data are represented as mean ± SD (*n* = 3); ** corresponds to *p* < .01.

The Na^+^/K^+^-ATPase enzyme on RBCs membrane plays a vital role in maintaining the morphology, structure and function of RBCs. It is mainly involved in transmembrane transport to maintain the balance of Na^+^ and K ^+^ inside and outside the cell (Mokrushnikov et al., 2015). As shown in Figure 4, compare with NRBCs, the Na^+^/K^+^-ATPase activity of BH-RBCs obtained after the drug-loading process *in vitro* was reduced from 30.87 ± 2.21 mmolpi·gHb^−1^·h^−1^ to 21.30 ± 1.49 mmolpi·gHb^−1^·h^−1^, which kept about 69% of NRBCs activity on the NRBCs membrane. The results indicated that the drug-loading process *in vitro* has a little effect on the activity of Na^+^/K^+^-ATPase of the RBCs. The decrease may be caused by multiple centrifugation, washing, and pre-swelling during the drug-loading process.

### 3.5. Phosphatidylserine exposure

**Figure 5.**
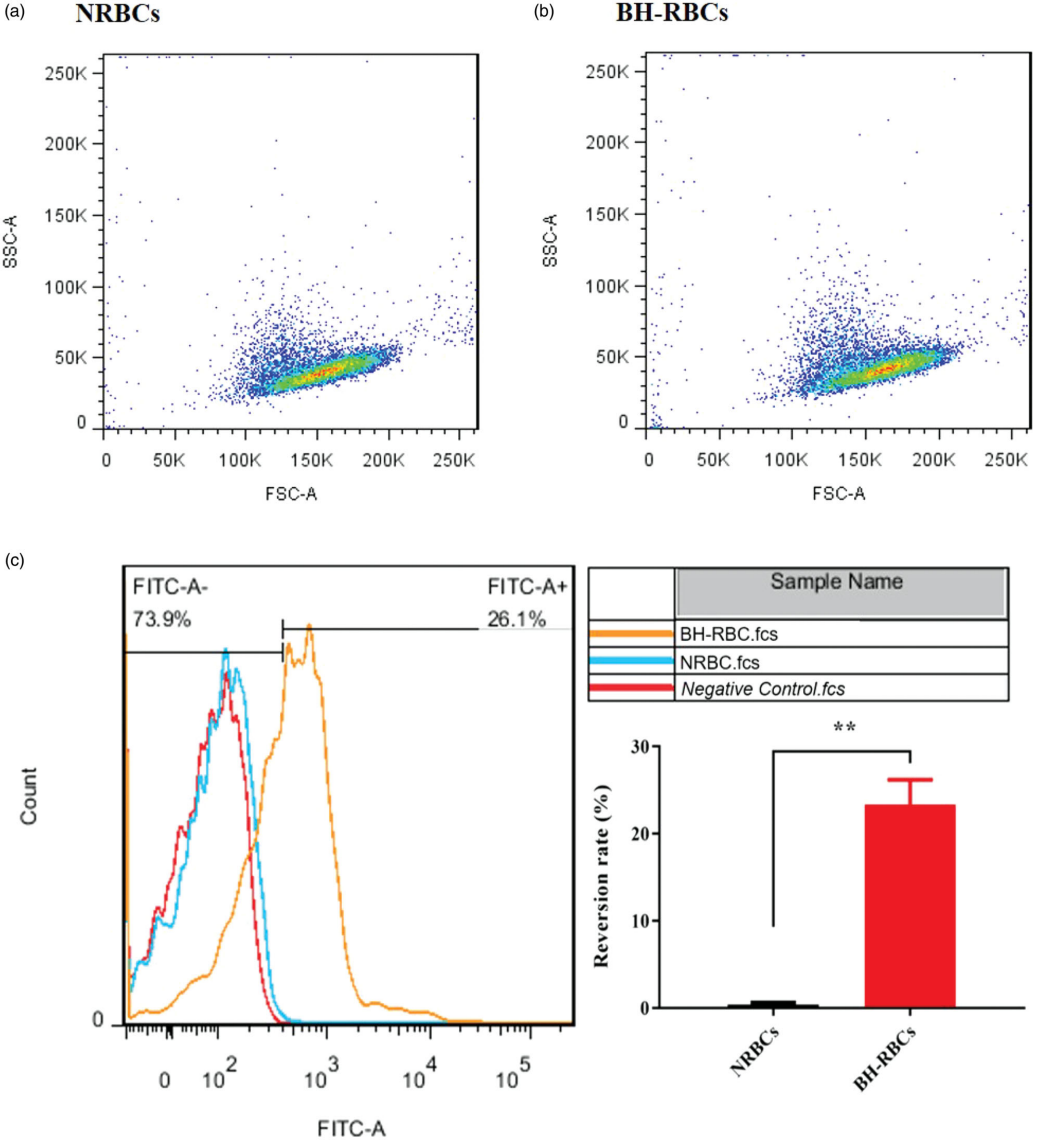
PS surface exposure of NRBCs and BH-RBCs. (A) & (B) FSC-SSC scatter plot; (C) PS reversion rate; ** corresponds to *p* < .01.

The presence of phosphatidylserine exposure in the outer lipid layer of RBCs membrane was analyzed by flow cytometric method. The FSC-SSC (forwad scatter- side scatter) results in Figure 5(a,b) show that NRBCs and BH-RBCs have the same distribution, indicating the similar morphology of BH-RBCs to NRBCs. PS externalization values of BH-RBCs after the loading process was about 26.1 ± 2.9% (Figure 5(c)), which displayed significant difference compared with NRBCs (0.4 ± 0.2%). Once PS is exposed overly, the RBCs were selectively recognized and ingested by PS receptors on the phagocytic cell membrane, which can predict that it has affected circulation time.

### 3.6. Survival of BH-RBCs

**Figure 6.**
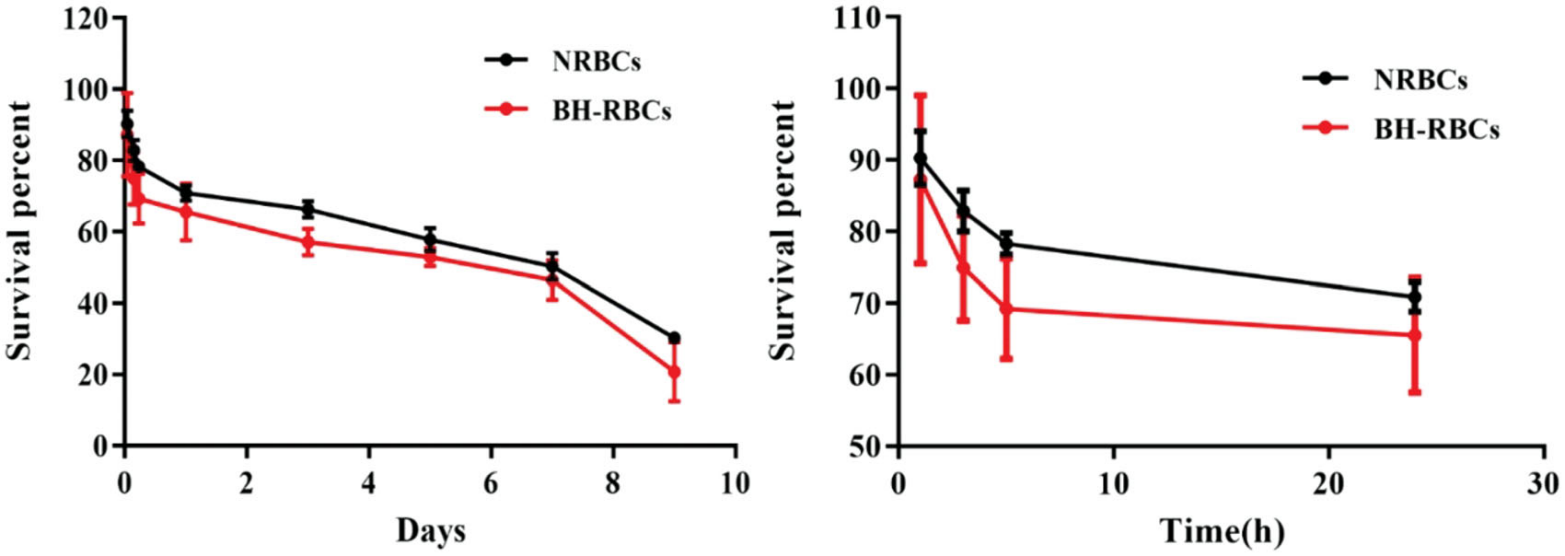
*In vivo* survival of NRBC and BH-RBCs. (A) Survival in 9 d; (B) survival in the first 24 h. Data are represented as mean ± SD (*n* = 3).

The cells with a higher degree of damage can be cleared from the circulation more easily, while cells with less damage can be circulated in the blood longer. We plotted the survival curves of RBCs in each group after 9 d of reinfusion. The results in Figure 6 show that after NRBCs were administered to the rat, it was rarely cleared within the first 24 h and the clearance rate of BH-RRCs was about 35%. A certain gap between the lifetime of NRBC and the theoretical value of RBC was observed, which may be due to a series of *in vitro* operations such as centrifugation, biotin treatment, reinfusion treatment and exposure to *in vitro* for a certain period. After 9 d of RBCs reinfusion into rats, the survival rate of NRBCs and BH-RBCs group was 30.29% and 20.74%, indicating that the autologous drug-loaded RBCs was survived for at least 9 d, which proved the potential of long circulation.

### 3.7. Pharmacokinetics (PK) study

**Figure 7.**
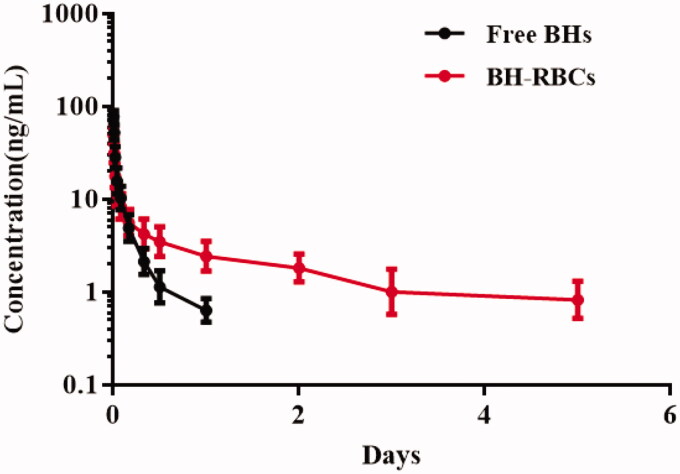
Pharmacokenicts of NRBCs and BH-RBCs. Data are represented as mean ± SD (*n* = 6).

The PK properties of BH released from the BH-RBCs and BH solution were determined in rats (*n* = 6). As shown in Figure 7, the content of BH in plasma from 0.5 ng/mL to 100 ng/mL could be calculated by the UPLC-MS method. The results showed that the release of free BH was rapid after the single vein injection, and it almost could not be detected after 24 h. However, the BH plasma concentration of the BH-RBCs group could be detected after 120 h. As shown in Table 1, the half-life (*t*_1/2_) of BH in BH-RBCs preparations was 1.97 d, which indicated a sustained release effect compared with free BH preparation with *t*_1/2_ of 0.42 d. Therefore, the BH loaded into RBCs could significantly improve the sustained release characteristic and prolong the circulation time.

**Table 1.** Summary of PK parameters for BH preparations.

Preparations	*T*_1/2_ (d)	*C*_max_ (ng/mL)	AUC_0–t_ (ng/mL*d)	AUC_0–∞_ (ng/mL*d)
Free BHs	0.42 ± 0.13	78.34 ± 12.51	4.07 ± 1.26	4.45 ± 1.35
BH-RBCs	1.97 ± 0.62	48.75 ± 10.58	10.38 ± 3.29	12.75 ± 3.46

### 3.8. Safety test

**Figure 8.**
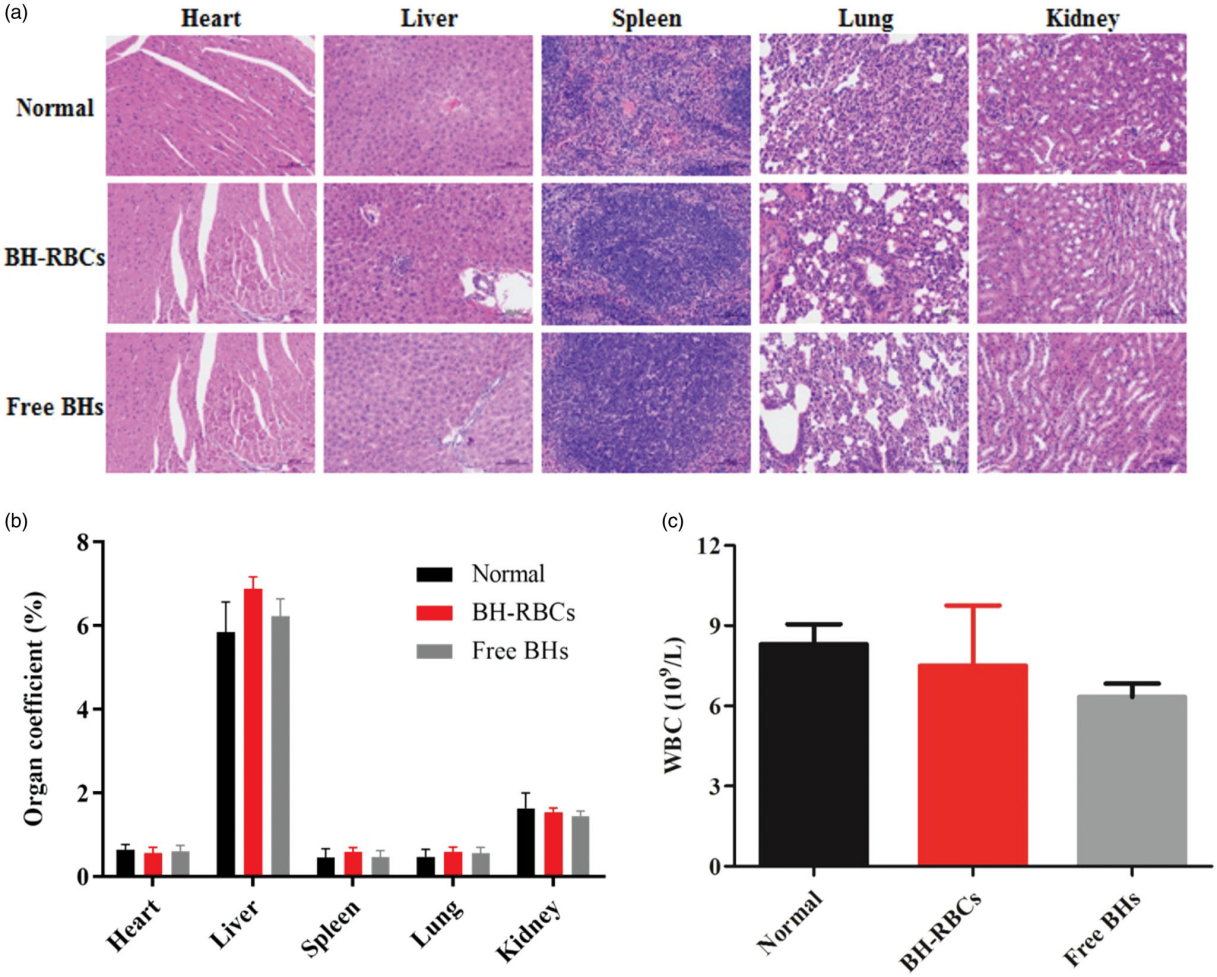
Safety test of all BH preparations. Data are represented as mean ± SD (*n* = 6).

After 15 d of the administration, the conditions of mice in each group were observed. There were no abnormalities of mice in activity, coat color, eating, drinking, etc., and no symptoms of poisoning & death. Meanwhile, the results in Figure 8 showed that HE staining of various tissues and blood routine conditions (white blood cell, WBC) in the BH-RBCs group and the free BH group almost has no difference with normal group, indicating that BH-RBCs did not cause acute toxic effects with a certain degree of safety.

### 3.9. *In vivo* determination of the hypolipidemic effect

**Figure 9.**
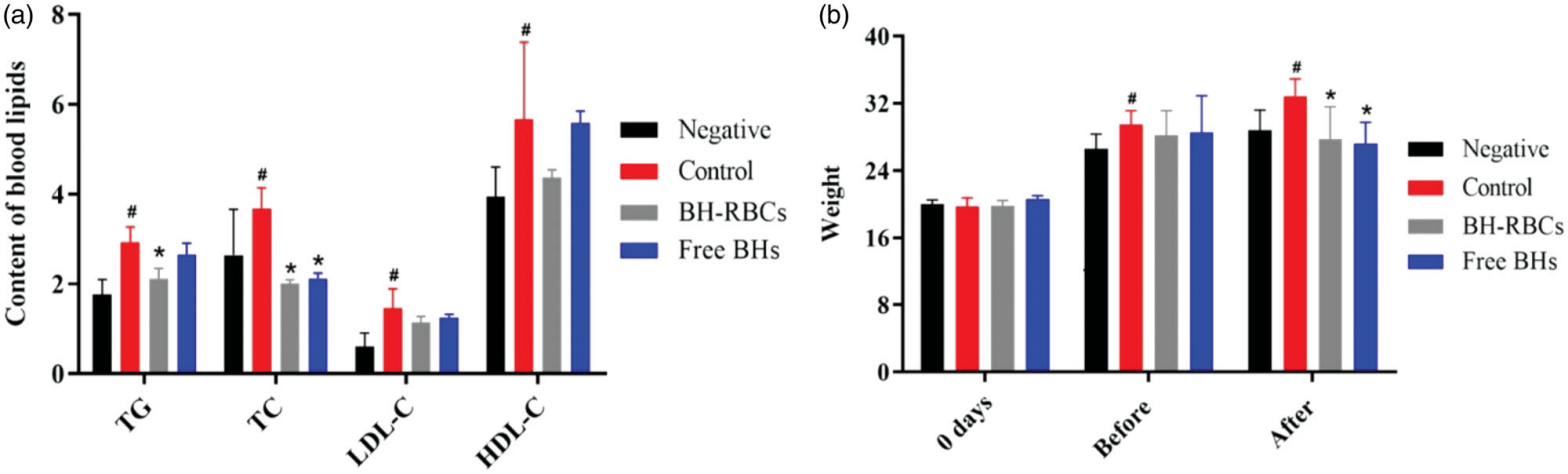
Summary of hypolipidemic effect for different preparations (mean ± SD, *n* = 6). Remarks: “Before” means the time from modeling to administration; “After” means the time after administration; # indicates a significant difference compared with the negative group (*p* < .05); * indicates a significant difference compared with the control group (*p* < .05).

The level of TG, TC, LDL-C and HDL-C was measured before and after the administration (Zhao et al., 2017). The results in Figure 9 shows that after modeling, the average levels of TG, TC, LDL-C and HDL-C in the control, BH-RBCs and free BH group were higher than those in the negative group (*p* < .05), indicating the hyperlipidemia model of mice was successful. After one month of administration, compared with the control group, the level of TG, TC, LDL-C (*p* < .05) was significantly reduced in BH-RBCs preparation. The level of TG, TC, LDL-C was also reduced in free BH group which was consistent with the literature report, but the effect was not significant as BH-RBCs preparation and had no considerable significance with the control group. The results showed that the BH-RBCs preparation has a prolonged effect compared with the free BH preparation. Thus, it has a significant hypolipidemic effect in the mouse hyperlipidemia model.

## 4. Conclusions

In summary, a new BH-RBCs delivery system has been developed to prolong the drug release and increase its biocompatibility simultaneously. The *in vivo* results indicated that the new delivery system has achieved the long-acting effect, although the decreased activity of ATPase and more exposure of PS proved the damages to erythrocytes caused by loading procedure. The study has demonstrated the potential delivery system to solve problems of BH such as rapid metabolic *in vivo*, low bioavailability, and lack of sustained-release effect. And it will have the good prospect in the future.
